# Chronic and transient loneliness in western countries: risk factors and association with depression. A follow-up study.

**DOI:** 10.1192/j.eurpsy.2024.691

**Published:** 2024-08-27

**Authors:** J. Domènech-Abella, C. Domènech

**Affiliations:** ^1^Research, Parc Sanitari Sant Joan de Déu, Sant Boi de Llobregat; ^2^Mental Health, Hospital Sant Joan de Déu, Barcelona, Spain

## Abstract

**Introduction:**

While transient loneliness refers to feelings that last for a short time (less than two years), chronic loneliness alludes to feelings that last more than two years. Transient loneliness can appear after stressful life events such as retirement and loss of close social connections whereas chronic loneliness is more strongly related to maladaptive social cognition, poor social support, and lack of intimate relationships. In comparison to transient loneliness, chronic loneliness is more strongly linked to mental health problems, particularly the incidence and recurrence of depression. Therefore, understanding the specific risk factors for both types of loneliness would be of great utility in mitigating their impact on mental health.

**Objectives:**

Our aim was to test distinct measures and risk factors for chronic and transient loneliness as well as cross-sectional and longitudinal associations of transient and chronic loneliness with depression.

**Methods:**

Responses from participants in Wave 5 (T1, 2013) and Wave 6 (T2, 2015) of The Survey of Health, Ageing and Retirement in Europe (SHARE) (N=45,490) were analyzed. The existence of clinically significant symptoms of depression was defined as reporting a value≥4 on the Euro-D scale. Loneliness was measured through 3-item loneliness scale and a single question. Both measures were tested in separate logistic regression models to identify risk factors for transient (loneliness at T1 but not at T2) and chronic loneliness (loneliness at both time points) as well as their impact on depression.

**Results:**

Between 47% and 40% of the cases of loneliness became chronic, according to the UCLA scale and the single question, respectively. Risk factors for both loneliness courses were being female, not being married, having a low educational level, having a poor physical health, having a poor social network and living in a culturally individualistic country. Risk factor for chronic loneliness were stronger, particularly those related to health status and social networks. Chronic loneliness showed also a strong association with depression both cross-sectionally and longitudinally, while transient loneliness showed a weaker cross-sectional association and markedly lower probabilities in the longitudinal association.

**Conclusions:**

Risk factors for chronic loneliness and measures of the temporal dimension of loneliness should be considered in psychosocial interventions designed to prevent mental disorders.

**Disclosure of Interest:**

None Declared

